# Physical Activity, Body Mass Index, and Cardiorespiratory Fitness among School Children in Taiwan: A Cross-Sectional Study

**DOI:** 10.3390/ijerph110707275

**Published:** 2014-07-16

**Authors:** Pei-Lin Hsieh, Min-Li Chen, Chiu-Mieh Huang, Wen-Chyuan Chen, Chun-Huei Li, Li-Chun Chang

**Affiliations:** 1 School of Nursing, Chang Gung University of Science and Technology, 261 Wen-Hua 1st Road, Gui-Shan Town, Tao-Yuan County 333, Taiwan; E-Mails: plhsieh@gw.cgust.edu.tw (P.-L.H.); mlchen@gw.cgust.edu.tw (M.-L.C.); 2 School of Nursing, National Yang-Ming University, 155 Li-Nong Street, Taipei 11221, Taiwan; E-Mail: cmhuang@ym.edu.tw; 3 Senior Exercise and Health Sciences Research Center, Chang Gung University of Science and Technology, 261 Wen-Hua 1st Road, Gui-Shan Town, Tao-Yuan County 333, Taiwan; E-Mail: kennycwc@gmail.com; 4 Guailin Elementary School, 82 Shulin, Budai Village, Budai Town, Chiayi County 625, Taiwan; E-Mail: lch1120@pchome.com.tw

**Keywords:** children, obesity, cardiorespiratory, health promotion, school nursing

## Abstract

There is evidence that cardiorespiratory fitness and physical activity significantly reduce cardiovascular risks in adults. A better understanding of the association between cardiorespiratory fitness, physical activity, and childhood obesity is vital in assessing the benefits of interventions to prevent obesity. This study was to examine the relationship between physical activity, body mass index, and cardiorespiratory fitness levels in Taiwanese children. A cross-sectional study was designed. Study participants consisted of 2419 school children (1230 males and 1189 females) aged 12 years old living in a southern Taiwan county with one the highest countrywide rates of childhood obesity. The weight status of the participants was defined as underweight, normal, overweight, or obese according to specific criteria. Cardiorespiratory fitness was then assessed by an 800-m run. Participants were queried on their physical activity habits via a questionnaire survey. The overall prevalence of overweight/obesity was 29.6%. Normal, underweight and overweight boys and girls had an increased odds ratio of being categorized with higher cardiorespiratory fitness than obese one for both gender. A significantly higher level of cardiorespiratory fitness was found in children who engaged in regular physical activity than in children who engaged only in irregular physical activity. Obese children are more likely to lack cardiorespiratory fitness. Physically active children have significantly better cardiorespiratory fitness levels than inactive children. This study supports the conclusion that BMI and physical activity are significantly correlated with cardiorespiratory fitness levels. Findings may provide educational professionals with information to assist their developing effective health promotion programs to healthy weight and improving cardiorespiratory fitness for children.

## 1. Introduction

The prevalence of childhood obesity is a major public health issue and has been increasing dramatically over the last decades [1,2]. In Taiwan, a nutritional survey of the nation between 1997 and 2002 revealed that the overall prevalence of overweight school children was 29% in boys and 21% in girls [3]. The prevalence rates of childhood obesity in Taiwan are higher than in Hong Kong (China) and Japan [4,5,6]. The highest rate of overweight and obese children (29.7%) was found in a southern Taiwan county in 2007 [7].

Epidemiological research has demonstrated that obesity and poor cardiorespiratory fitness performance contribute significantly to the prevalence of cardiovascular disease (CVD); in addition, related factors have been found to stem from childhood into adulthood [8]. Childhood obesity is also a significant risk factor for some cancers and type 2 diabetes mellitus (formerly known as non-insulin-dependent diabetes) in adulthood [9,10]. Children with high cardiorespiratory fitness and low body mass index (BMI) have a lower metabolic syndrome risk compared to those with low cardiorespiratory fitness and high BMI [11,12]. Studies show that health professionals need to encourage better fitness and resolve obesity-related problems in children to ensure overall positive health during their childhood and into adulthood.

As shown in previous studies [13,14], gender influences the association between cardiorespiratory fitness and childhood obesity. Health professionals should design programs to address childhood obesity by recognizing the correlation between gender, BMI and cardiorespiratory fitness, especially in geographical areas with a high prevalence of obesity. These would help alleviate chronic diseases and future problems caused by obesity. 

Physical activity is an important for improving cardiorespiratory fitness. Several studies have demonstrated that more active children have better cardiorespiratory fitness than inactive ones [15,16,17]. Further, studies have found children who do regular physical activity have greater muscle strength [18] and flexbility [19] than children who do not do regular physical activity. These findings suggest a link between physical activity and physical fitness, particularly in improving cardiorespiratory fitness. 

Physical fitness in particular cardiorespiratory fitness is an important theme of the health promoting schools program issued by the Ministry of Education Taiwan [20], the cardiorespiratory fitness was integrated into the physical activity classes. Cardiorespiratory fitness is assessed in all elementary schools for children aged 12 years old. Cardiorespiratory fitness in Taiwan is normally assessed using the 800-m run test, and the maximum volume of oxygen (*V*O_2max__) _can be estimated from a score obtained after this test from equations [20]. According to Wu [21], the 800-m run significantly improves the correlation between* V*O_2max_ and running performance. These test results show high correlations with *V*O_2max_ and Her [22] subsequently obtaining a correlation of 0.81 and 0.77, as indicated by Tsai and Yung [23] in a study of 12 year-old school children. Based on those results, Taiwanese health professionals in schools use the 800-m run test as a predictor of *V*O_2max_ for school children. 

Rapid increases in obesity and decreased physical fitness levels, particularly in cardiorespiratory fitness among Taiwanese children, warrant an investigation into how weight status and physical outcomes are related in Taiwanese children [24]. Therefore, this study examines the relationship between physical activity, BMI (underweight, normal, overweight and obese) and cardiorespiratory fitness levels in Taiwanese children. 

## 2. Experimental Section

### 2.1. Design and Sampling

The Ethical Review Committee of Chang Gung Hospital (Linkou, Taiwan) approved this study (No. 100-3525B). Both parents and students were oriented on the objectives and procedures of this study, upon which, parents or guardians and students gave their written informed consent before they participated in the study. 

A cross-sectional study was designed. Participants were recruited from all elementary schools in a southern Taiwan county with one the highest rates of childhood obesity countrywide. Children aged 12 years old were invited to participate in this study. Some children refused to complete the questionnaire, and incomplete questionnaires were excluded. During the period from November 2010 to April 2011, we recruited 2514 children in this study, and a final number of 2419 children (1230 male, 1189 female) completed the program with 96.2% response rate.

### 2.2. Instruments

#### 2.2.1. Anthropometric Measurements

Height and weight of barefooted participants in light clothing were measured in the first semester in November 2010. All measurements were taken by trained physical education teachers following standard operating procedures. Teachers calculated BMIs by dividing weight by the square of the participants’ height (BMI = weight (kg)/height (m)^2^). Participants were classified based on the BMI international cutoff values as underweight, normal weight, overweight, or obese [25,26].

#### 2.2.2. Physical Activity

A self-administered habitual physical activity questionnaire was obtained and participants completed the questionnaire with the instruction of trained school teachers. Participants were asked to recall and record activities including frequency and duration of indoor/outdoor activities. The questionnaire was established by the John Tung Foundation [27], and has been found to be validated and has acceptable test/retest reliability by the Ministry of Education, Taiwan [20]. Participants were asked the question, “How many times weekly do you engage in physical sports outside of school (jogging/running, gymnastics, dancing, swimming, ball games and free play, *etc.*)?” Physical sports were defined as moderate and/or vigorous physical activities in the questionnaire. The possible answers were none, 1–2 times, 3–4 times, 5–6 times, and more than 7 times. Another question was, “How many minutes daily do you engage in physical sports outside of school?” The possible answers were less than 15 min, 16–30 min, 31–15 min, 46–60 min, and more than one hour. Participants who participated in sports and/or vigorous activity at least three times weekly with each session lasting at least 30 min were defined as having regular physical activity. Irregular physical activity was defined as participants who participated in sports and/or vigorous free play less than three times per week for less than 30 min each time. Their physical activity status was dichotomized into regular physical activity and irregular physical activity. 

#### 2.2.3. Cardiorespiratory Fitness

Cardiorespiratory fitness was assessed by an 800-m run held during physical education classes in elementary schools. The 800-m run was measured once to evaluate the speed of movement. Children were tested in groups of 6. Children were instructed to maintain a steady pace and finish as quickly as possible. If unable to continue running, the students were allowed to walk. Times were recorded in seconds. Physical education teachers in each school took the measurements. Protocols were described in detail in a technical manual provided to all schools and participating teachers, as well as demonstrated and practiced at local and regional training seminars. All timekeepers used in this study were calibrated regularly. Quality control and safety during the measurements were emphasized. 

Participant performances in the cardiorespiratory test were classified into one of five levels, based on the normal distribution of physical fitness in Taiwan [20]. The participants in the extreme qualities within cardiorespiratory performance were classified as performing poorly (*i.e.*, the lowest quartile less than 25th percentile) and performing well (*i.e.*, the highest quartile more than 75th percentile).

### 2.3. Data Analysis

First, descriptive statistics for gender, BMI and habitual physical activity were calculated to characterize all of the participants. All participants were analyzed with respect to gender difference in BMI, 800-m run score, BMI category and physical activity level with independent sample *t*-tests and chi-square tests. Second, participants in the “unfit” (*i.e.*, the lowest quartile) and “fit” (*i.e.*, the highest quartile) groups [28] were selected for comparison of the differences in BMI categories and physical activity levels between the genders with chi-square tests. The association between BMI, physical activity levels, and cardiorespiratory fitness was also analyzed using binary logistic regression. The dummy variable for weight status was used. Obese students with irregular physical activity were used as the reference groups. Next, confidence intervals (CIs) were calculated. All analyses were performed based on gender. Analyses were performed with SPSS version 15.0 (SPSS, Inc., Chicago, IL, USA) and the level of significance was set to 0.05. 

## 3. Results

Table 1 summarizes the prevalence of overweight and obese participants by gender. The prevalence of overall combined overweight and obese children was 29.6%. Boys were more likely to be overweight/obese than girls (34.0%* vs.* 25.0%). Prevalence of underweight participants was 16.0% in boys and 16.2% in girls. Overall, 53.9% of the participants had irregular physical activity, while 46.1% had regular physical activity. Boys performed significantly (*p* < 0.001) better in the 800-m run (276.65* vs.* 287.13 seconds) than girls did. 

**Table 1:** Sample characteristics.

Characteristics	Boys (*n* = 1230)	Girls (*n* = 1189)	Total (*n* = 2419)	*p*-value
BMI (mean and SD)	20.48 (4.47)	19.68 (3.69)	20.09 (4.12)	<0.001
800-m run (s)	276.65 (64.41)	287.13 (56.90)	281.80 (61.05)	<0.001
BMI category (*n* and %)				<0.001
Underweight	197 (16.0)	195 (16.4)	392 (16.2)	
Normal weight	614 (49.9)	697 (58.6)	1,311 (54.2)	
Overweight	170 (13.8)	144 (12.1)	314 (13.0)	
Obesity	249 (20.2)	153 (12.9)	402 (16.6)	
Physical activity (*n* and %)				<0.001
Regular	699 (56.8)	416 (35.0)	1,115 (46.1)	
Irregular	531 (43.2)	773 (65.0)	1,304 (53.9)	

Table 2 shows the characteristics of the participants in the fit and unfit categories. A total of 1260 elementary school children (628 boys, 632 girls) were categorized as unfit (*i.e.*, the lowest quartile) and fit (*i.e.*, the highest quartile). The proportions of overweight/obese-unfit boys were significantly higher than that of overweight/obese-fit ones (16.2%* vs.* 6.1%; 41.5%* vs.* 3.4%). The proportion of overweight/obese-unfit girls was significantly higher than that of overweight/obese-fit ones (14.1%* vs.* 9.7%; 22.3%* vs.* 5.3%). The fit group for both genders had significantly higher rates of regular physical activity than the unfit group for both genders (boys: 71.6%* vs.* 48.6%; girls: 42.6%* vs.* 30.0%). 

Table 3 shows the results of binary logistic regression analysis on cardiorespiratory fitness, based on BMI and with regular/irregular physical activity for boys and girls. Underweight children were more likely to belong to the fit group than the obese children were (boys: Odds ratio (OR): 19.29, 95% CI = 9.29–40.05; girls: OR: 6.24, 95% CI = 3.24–12.04). Additionally, the normal weight children were more likely to belong to the fit group than the obese children were (boys: OR: 19.75, 95% CI = 10.21–38.22; girls: OR: 4.66, 95% CI = 2.69–8.09). Moreover, the overweight children were more likely to belong to the fit group than the obese children were (boys: OR: 3.24, 95% CI = 1.40–7.49; girls: OR: 2.68, 95% CI = 1.34–5.33). 

**Table 2:** Characteristics of the participants in the extreme qualities based on gender and cardiorespiratory fitness.

Measure	Boys (*n* = 628) Unfit	Fit	*p*-value	Girls (*n* = 632) Unfit	Fit	*p*-value
Weight status (*n* and %)			<0.001			<0.001
Underweight	41 (11.3)	65 (24.6)		36 (11.5)	64 (20.1)	
Normal weight	113 (31.0)	174 (65.9)		163 (52.1)	207 (64.9)	
Overweight	59 (16.2)	16 (6.1)		44 (14.1)	31 (9.7)	
Obesity	151 (41.5)	9 (3.4)		70 (22.3)	17 (5.3)	
Physical activity			<0.001			<0.001
Regular	177 (48.6)	189 (71.6)		94 (30.0)	136 (42.6)	
Irregular	187 (51.4)	75 (28.4)		219 (70.0)	183 (57.4)	

Notes: Weight status is based on the BMI international cutoff values as underweight, normal weight, overweight and obese. Regular physical activity is defined as children who participated in sports and/or vigorous free play at least three times per week for at least 30 min each time. Irregular physical activity is defined as children who participated in sports and/or vigorous free play less than three times per week for less than 30 min each time.

**Table 3:** Cardiorespiratory fitness indicators in boys and girls.

Measure	Boys (*n* = 628) OR	95% CI	*p*-value	Girls (*n* = 632) OR	95% CI	*p*-value
BMI (Obesity)						
Underweight	19.29	9.29–40.05	<0.001	6.24	3.24–12.04	<0.001
Normal weight	19.75	10.21–38.22	<0.001	4.66	2.69–8.09	<0.001
Overweight 3.24	3.24	1.40–7.49	0.006	2.68	1.34–5.33	0.005
Physical activity (Irregular)						
Regular	2.05	1.40–3.01	<0.001	1.63	1.16–2.30	<0.001

Notes: OR, odds ratio; CI, confidence interval. Weight status is based on the BMI international cutoff values as underweight, normal weight, overweight and obese. Regular physical activity is defined as children who participated in sports and/or vigorous free play at least three times per week for at least 30 min each time. Irregular physical activity is defined as children who participated in sports and/or vigorous free play less than three times per week for less than 30 min each time.

Although the children with regular physical activity were more likely to belong to the fit group than the children with irregular physical activity were (boys: OR: 2.05, 95% CI = 1.40–3.01; girls: OR: 1.63, 95% CI = 1.16–2.30), active boys had higher odds than active girls. Regular physical activity plays an important role in cardiorespiratory fitness.

## 4. Discussion

This study examined the relationship between physical activity, BMI (underweight, normal, overweight and obese) and cardiorespiratory fitness levels in Taiwanese children. According to our results, the prevalence of overweight and obese children for both boys and girls (boys: 34.0%; girls 25.0%) is significantly higher than that found in the national survey in Taiwan (boys: 29%; girls: 21%) [7]. The prevalence of overweight and obese children in our study are much higher (boys: 34.0%; girls 25.0%) than that in China (boys: 26.5%; girls: 18.7%) [5] and Japan (boys: 17.6%; girls 12.2%) [4]. Similarly, Chu and Pan [29] evaluated the prevalence of obesity among 2450 elementary schoolchildren and found that the highest rates occurred in southern Taiwan. Our results show that the childhood obesity trend in southern Taiwan is generally higher than in other Asian countries. 

Another interesting result was that the prevalence of underweight children in our study (boys: 16.4%; girls 16.0%) was higher than that in China (boys: 2.0%; girls 4.7%) (Shang* et al.* [6]) in Japan (about 3% for both genders) [30], or in Portugal (boys: 3.9%; girls 5.6%) [31]. Our study shows that southern Taiwan generally had more underweight and overweight children than the northern and central areas of the country. This may be related to socio-economic status. The participants in this study were selected from suburban areas of Taiwan where approximately 10% of children are commonly reared by their grandparents and are from lower socioeconomic groups [32]. As shown in previous studies [33,34,35], lower levels of education and lower income levels are associated with being underweight, overweight and obese. Further studies including socioeconomic data would help elucidate these complex relationships.

Our results indicate that Taiwanese children of a normal BMI generally have a better cardiorespiratory fitness level than underweight and overweight/obese children. He* et al.* [36] demonstrate strong negative association between cardiorespiratory fitness levels and Chinese children’s BMI and weight gain. An impaired running ability, often observed in obese children, is closely related to the increased demand required to move the excess body weight [37,38,39]. Therefore, further research is warranted, especially longitudinal studies of cardiorespiratory fitness levels and body size.

This study has demonstrated that physically active children have a significantly higher cardiorespiratory fitness level than inactive children do. Ruiz* et al.* [40] observed Spanish youth and came to a similar conclusion. Cardiorespiratory fitness has been considered a better index of the activity level than direct and short-term measures of physical activity [18]. Although our results cannot be compared with international studies due to using different methods, these results could support a recommendation to the government to develop health promotion strategies that target cardiorespiratory fitness for children. 

### Limitations

Despite its contributions, this study has several limitations. The results of this study regarding relationship between physical activity, body mass index, and cardiorespiratory fitness levels in Taiwanese children should be interpreted carefully. The cross-sectional design of this study cannot be drawn conclusions on the direction of the relationships. Results can be only generalized to similar populations. Another limitation relies on the self-reported of physical activity. Cardiorespiratory fitness was not assessed by the *V*O_2max_ response which is an important determinant of the aerobic contribution. However, previous studies [22,23,41] have demonstrated a high correlation with 800-m run test. Sexual maturation of the girls was not assessed. Maturing girls may perform poorer in physical fitness tests due to higher fat mass [42,43]. Finally, this study classified weight status based on BMI. Despite its extensive use to define weight status, BMI is not an accurate measure of fat. Percentage of body fat should be considered, and precise measures of body fat should be taken in future studies. 

## 5. Conclusions

The overall prevalence of overweight and obese children in southern Taiwan is higher than in other Asian countries. Also, boys performed significantly better on cardiorespiratory fitness tests than girls did. Additionally, BMI significantly influences cardiorespiratory fitness levels for both boys and girls. This study also finds that children who are physically active have a significantly higher cardiorespiratory fitness level than those who are inactive. Given the high prevalence of childhood obesity, improving the cardiorespiratory fitness level of children could dramatically improve public health. Further studies should elucidate such complex relationships by incorporating a level of physical activity and including data on dietary intake, puberty, and socioeconomic status. 
